# The importance of being parasiticidal… an update on drug development for the treatment of alveolar echinococcosis

**DOI:** 10.1016/j.fawpar.2019.e00040

**Published:** 2019-03-14

**Authors:** Britta Lundström-Stadelmann, Reto Rufener, Dominic Ritler, Raphael Zurbriggen, Andrew Hemphill

**Affiliations:** Institute of Parasitology, Department of Infectious Diseases and Pathobiology, Vetsuisse Faculty, University of Bern, Länggassstrasse 122, 3012 Bern, Switzerland

**Keywords:** AE, alveolar echinococcosis, SPEMs, small particles of *Echinococcus multilocularis*, ABZ, albendazole, MBZ, mebendazole, PGI, phosphoglucose isomerase, MMV, Medicines for Malaria Venture, 2-ME, 2-methoxyestradiol, MAPK, mitogen activated protein kinases, *Echinococcus multilocularis*, Drug treatment, Chemotherapy, Albendazole, Mefloquine, Malate dismutation

## Abstract

The lethal disease alveolar echinococcosis (AE) is caused by the metacestode stage of the fox tapeworm *Echinococcus multilocularis*. Current chemotherapeutical treatment of AE relies on albendazole and mebendazole, with the caveat that these compounds are not parasiticidal. Drugs have to be taken for a prolonged period of time, often life-long, which can cause adverse effects and reduces the patients' quality of life. In some individuals, benzimidazoles are inactive or cause toxicity, leading to treatment discontinuation. Alternatives to benzimidazoles are urgently needed. Over the recent years, *in vivo* and *in vitro* models for low-to-medium throughput drug discovery against AE have been set in place. *In vitro* drug tests include the phosphoglucose-isomerase (PGI) assay to measure physical damage induced to metacestodes, and viability assays to assess parasiticidal activity against metacestodes and stem cells. *In vitro* models are also employed for studies on mechanisms of action. *In vivo* models are thus far based on rodents, mainly mice, and benefits could be gained in future by comparative approaches in naturally infected dogs or captive monkeys.

For the identification of novel drugs against AE, a rare disease with a low expected market return, drug-repurposing is the most promising strategy. A variety of chemically synthesized compounds as well as natural products have been analyzed with respect to *in vitro* and/or *in vivo* activities against AE. We here review and discuss the most active of these compounds including anti-infective compounds (benzimidazoles, nitazoxanide, amphotericin B, itraconazole, clarithromycin, DB1127, and buparvaquone), the anti-infective anti-malarials (artemisinin, ozonids, mefloquine, and MMV665807) and anti-cancer drugs (isoflavones, 2-methoxyestradiol, methotrexate, navelbine, vincristine, kinase inhibitors, metallo-organic ruthenium complexes, bortezomib, and taxanes). Taking into account the efficacy as well as the potential availability for patients, the most promising candidates are new formulations of benzimidazoles and mefloquine. Future drug-repurposing approaches should also target the energy metabolism of *E*. *multilocularis*, in particular the understudied malate dismutation pathway, as this offers an essential target in the parasite, which is not present in mammals.

## Highlights

• Benzimidazoles are used to treat AE, but new drugs are needed.
• New drugs against AE can be identified by drug repurposing.
• Drugs against other infectious diseases and cancer can be repurposed against AE.
• Most promising are new formulations of benzimidazoles and mefloquine.
• Future approaches should include targeting the energy metabolism of the parasite.

## 1 Introduction

### 1.1 *Echinococcus multilocularis* and alveolar echinococcosis

Larval stages of the genus *Echinococcus* (Cestoda, Platyhelminthes) cause life-threatening diseases affecting humans and livestock. *Echinococcus granulosus sensu lato* and *E*. *multilocularis* (also known as the small fox tapeworm) are the two most prominent members of the genus. *E*. *granulosus* is found worldwide, inflicting considerable medical and economic constraints, mostly in resource-poor countries. On the other hand, *E*. *multilocularis*, the topic of this review, is distributed all over the Northern hemisphere. Highly endemic regions are located in Western-Central Europe (classically Switzerland, Southern Germany, Eastern France, and Western Austria), in Eastern-Europe including the Baltic countries, in Central and Western China, in Russia, in Hokkaido (Japan), and in Alaska (North-America) (Deplazes et al., 2017). Infection with *E*. *multilocularis* metacestodes causes the disease alveolar echinococcosis (AE) in humans, but also in dogs, captive monkeys, beavers, and other species (Deplazes and Eckert, 2001). 91% of human AE cases are found in the Tibetan plateau of Western China (Kern et al., 2017). However, AE is considered to be an emerging disease in many areas of the world, including Europe (Gottstein et al., 2015), Canada (Trotz-Williams et al., 2017), and particularly in Kyrgyzstan where AE has very recently become an increasing health problem (Bebezov et al., 2018; Deplazes et al., 2017). In Western-Central Europe, 0.3 to 3 per 1,000,000 inhabitants get infected with *E*. *multilocularis* annually and case numbers are on the rise (Gottstein et al., 2015). Due to its relatively rare abundance, investments in the development of new drugs against AE will not result in a high market return, and thus for many years the pharmaceutical industry has not been compelled to develop novel drugs against alveolar and cystic echinococcosis.

### 1.2 The biological features of *E*. *multilocularis*

*E*. *multilocularis* undergoes a typical predator-prey life cycle, involving a carnivorous definitive host and mostly small rodents as intermediate hosts. Humans, as well as dogs, beavers, captive monkeys, and others act as accidental intermediate hosts. Adult stages of the tapeworm live in the intestine of red foxes, arctic foxes, coyotes, raccoon dogs, wolves, or domestic dogs (Romig et al., 2017). Here the adult tapeworms undergo sexual reproduction and will produce infective eggs, which are released into the environment *via* fecal shedding. Those eggs contain an oncosphere (the first larval stage) and they are orally infective for various intermediate hosts, such as predominantly voles (e.g. *Microtus arvalis* or *Arvicola terrestris*), but also other small mammals (Romig et al., 2017). Once eggs are ingested and pass the stomach, the oncospheres get activated and are set free in the intestine, where they penetrate the intestinal tissue, reach blood and lymphatic vessels, and finally invade the liver. There they develop into the metacestode (second larval stage). Metacestodes grow continuously and can undergo virtually unlimited proliferation, building up a mass of parasite tissue that is intermingled with host connective tissue and immune cells, forming a metacestode lesion that will persist within a host for a lifetime. After 2–4 months (in rodents) metacestodes produce brood capsules filled with protoscoleces. These are precursors of newly formed tapeworm heads. Once intermediate hosts or tissues containing metacestodes and protoscoleces are ingested by a canid definitive host, protoscoleces attach to the intestinal epithelium and develop into adult tapeworms, concluding the life cycle.

For *E*. *multilocularis*, humans are aberrant intermediate hosts in that protoscoleces development has only rarely been described. Human infection thus represents a dead-end in the life cycle. Nevertheless, humans can develop the disease AE. For treatments of AE, metacestodes in the liver, or rarely also in other locations, are targeted by chemotherapeutical and surgical approaches (Kern et al., 2017).

**Fig. 1:**
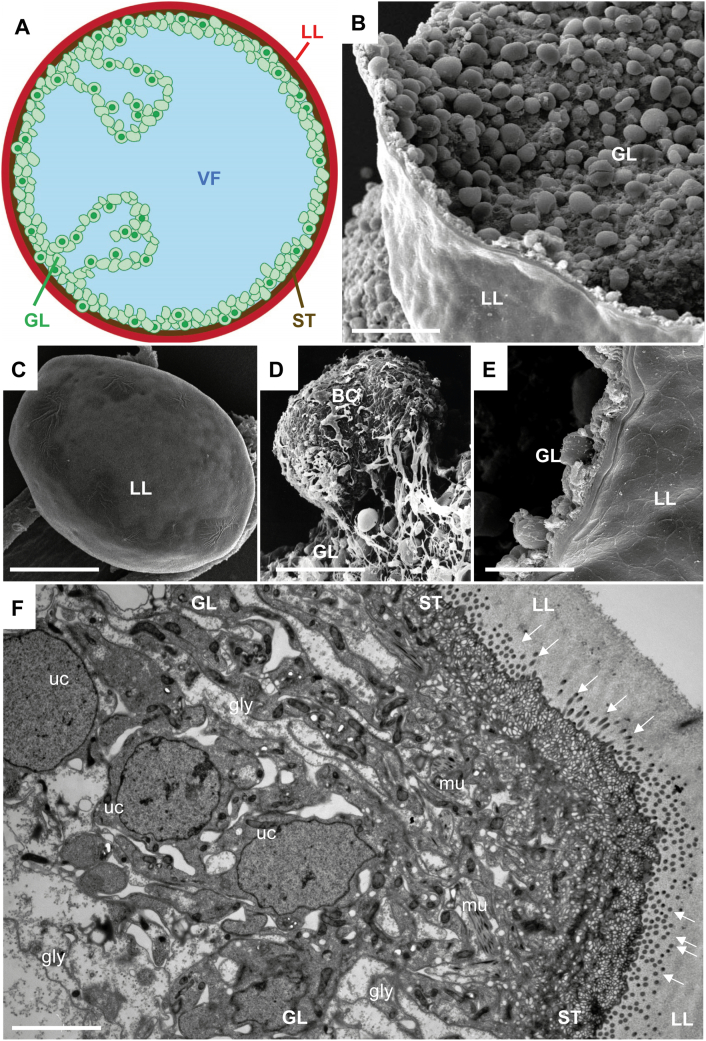
Structure of *E*. *multilocularis* metacestodes. (A) schematic view of a metacestode vesicle. The main components are color-coded: the laminated layer (LL, red); the syncytial tegument (ST, brown); the germinal layer (GL, green), the vesicle fluid (VF, blue). (B–E) Scanning electron micrographs (SEM) of *E*. *multilocularis* metacestodes. (B) View into the interior of a metacestode, showing the germinal layer (GL) and the outer laminated layer (LL). (C) Intact metacestode, with only the LL exposed. (D) Developing brood capsule (BC) still attached to the germinal layer (GL). (E) Higher magnification SEM image of the vesicle wall. (F) Section cut through the vesicle wall, shown by transmission electron microscopy (TEM). Note the outer laminated layer (LL), the syncytial tegument (ST) with microtriches protruding outwards into the LL (arrows), and the complex germinal layer (GL), containing undifferentiated cells (uc), muscle cells (mu), glycogen storing cells (gly), and also connective tissue. Bars in B = 330 μm; C = 1200 μm; D = 360 μm; E = 280 μm; F = 4.1 μm. (For interpretation of the references to color in this figure legend, the reader is referred to the web version of this article.)

Structurally, *Echinococcus* metacestodes are fluid-filled vesicles of a few millimeters in size, which exhibit a range of genus-specific features (see Fig. 1). The wall of these vesicles is divided into an inner germinal layer and an outer laminated layer. The acellular laminated layer is rich in carbohydrates and high molecular weight glycans, covers the entire metacestode surface, and mediates the direct physical contact with host immune and non-immune cells (Agudelo Higuita et al., 2016). Components that build up this laminated layer are produced and secreted by the germinal layer, which represents the live and metabolically active parasite tissue. Cells of the germinal layer also secrete vesicle fluid into the interior of metacestodes, and this vesicle fluid plays a role in nutrition and in exchange of metabolites within the parasite. The tegument, as a part of the germinal layer, mediates the direct association of the live parasite tissue with the inner surface of the laminated layer, and it is characterized by microvilli-like extensions named microtriches. The germinal layer is a complex tissue, and consists of muscle cells, nerve cells, glycogen storage cells, connective tissue cells, and totipotent stem cells (also called germinative cells or neoblasts) (Brehm, 2010a; Koziol et al., 2013, Koziol et al., 2014). Germinative cells make up 20–25% of all cells in the germinal layer. They are responsible for the high regenerative potential of the parasite, and they are suspected to be responsible for metastasis formation (Ali-Khan et al., 1983; Koziol and Brehm, 2015; Mehlhorn et al., 1983). *E*. *multilocularis* metacestodes reproduce asexually by exogenous formation and budding of daughter vesicles. The resulting lesions in an infected individual form a heterogeneous mass comprised of peripheral parasite tissue undergoing active proliferation, while the central part is often necrotized, probably due to the increased presence of host connective tissue that limits access of nutrients. Metastasis formation has been described, most likely mediated by germinative cells that are released into the environment of the parasite. This can affect neighbouring organs (gall bladder, abdominal lymph nodes, pancreas, diaphragm, and peritoneum), but also lungs, bones, and the brain, leading to severe complications in treatment (Kern et al., 2017). In addition, small particles of *E*. *multilocularis* (SPEMs) have been described, which appear as isolated particles of laminated layer throughout the liver of infected patients (Barth et al., 2012). They might function in parasite spread and/or immunomodulation.

### 1.3 Clinical hallmarks of AE and current treatment approaches

Infection with *E*. *multilocularis* is largely asymptomatic and remains often undiagnosed until a progressive state is reached. This time span can reach 10–15 years. At this advanced stage of disease, non-specific symptoms such as abdominal pain, jaundice, cholestasis, hepatomegaly, fever, anaemia, weight loss, and pleural pain occur (Kern et al., 2017). Diagnosis is based on non-invasive imaging techniques, serological tests, as well as specific staining techniques and PCR (Brunetti et al., 2010; Siles-Lucas et al., 2017).

Treatment of AE is based on surgical intervention and/or benzimidazole-based chemotherapy. Complete surgical resection is the only curative treatment, but is applied in only 20–50% of all cases (Brunetti et al., 2010; Kern et al., 2017). Unfortunately, radical surgery can often not be performed as most cases are diagnosed at the late stage of disease. A long-term cohort study in Germany showed that complete surgery could be performed in 16% and 36% of all AE patients (referring to cases described before and after the year 2000, respectively) (Grüner et al., 2017). If surgery is performed, it is always accompanied by benzimidazole treatment for at least 2 years thereafter, and monitoring of patients should be continued for 10 years (Brunetti et al., 2010). Inoperable cases of AE must undergo long-term/mostly life-long treatment with the benzimidazoles albendazole (ABZ) or mebendazole (MBZ). Recommended dosages are 10–15 mg ABZ/kg/day, in 2 doses. Alternatively, MBZ can be given at 40–50 mg/kg/day, divided into three doses (Brunetti et al., 2010).

Adverse reactions to long-term benzimidazole treatment such as severe hepatotoxicity may occur. To avoid such adverse effects, regular monitoring of liver enzymes, drug serum levels and, if necessary, adjustment of the dosage is needed. However, this is highly dependent on a health service with a functional infrastructure, which does not exist in all countries strongly affected by AE. A study with more than 3000 cystic echinococcosis patients treated with ABZ showed that gastrointestinal tract problems represent the most common adverse events, but no fatal cases were described (Horton, 1997). More recently, a long term study on AE patients carried out in Germany showed that 54.5% of all patients experienced mild side-effects, and 6.9% of the patients experienced life-threatening adverse effects such as hepatotoxicity that led to treatment-discontinuation (Grüner et al., 2017). Moreover, MBZ and ABZ may induce embryotoxic or teratogenic effects (Horton, 1989), and are not recommended to be used in pregnancy. Still, clinical studies showed that chemotherapy has significantly increased the 10-year survival rate from 6 to 25% to 80–85% for AE patients who could not undergo any or radical surgery (Grüner et al., 2017; Kern et al., 2017). A major setback of benzimidazoles is that they are not parasiticidal, but rather parasitostatic *in vivo* (Reuter et al., 2004), a problem which is further discussed in Section 1.4. Therefore, recurrence rates after treatment interruption or discontinuation are high, especially in patients not appropriately followed-up (Reuter et al., 2004; Stumpe et al., 2007). In up to 16% of all AE cases disease progression due to treatment failure was described (Ammann et al., 1994). In countries with well-developed health-care systems, including access to treatment and drug level-monitoring, a relatively good clinical management of AE can be achieved. AE is still a lethal disease in less developed countries with low, or limited, financial resources (Kern et al., 2017). In more industrialized countries, the costs for treating one AE patient sum up to over 100,000 Euros annually (Torgerson et al., 2008). Recent studies have also pointed out the reduction of health-related quality of life of AE patients (Schmidberger et al., 2018) and the increased psychological burden, including high levels of depression and anxiety, as well as reduced physical life quality (Nikendei et al., 2019).

Besides benzimidazoles, only two other compounds have been applied clinically against AE: amphotericin B and nitazoxanide (see also Section 3.1). The anti-fungal agent amphotericin B was applied in patients as a salvage treatment, but it was not parasiticidal, and upon long term usage induced nephrotoxicity (Tappe et al., 2009). Nitazoxanide is a broad spectrum anti-infective drug, which, despite promising activities in mouse studies, failed to be active against human AE (Kern et al., 2008; Tappe et al., 2009).

Taken together, the numbers of human AE cases are continuously rising, no alternative to benzimidazoles has been developed, and a high proportion of patients experiences life-threatening side-effects. Thus, new and better treatment options against AE are urgently needed.

### 1.4 Why are benzimidazoles not parasiticidal against *E*. *multilocularis?*

One possible explanation for the parasitostatic rather than parasiticidal activity of benzimidazoles against AE can be found at the molecular level: After oral ingestion, ABZ is converted into ABZ-sulfoxide (also called ricobendazole), and at a later stage further to ABZ-sulfone. The active metabolite ABZ-sulfoxide binds to a specific domain of the beta-tubulin subunit, impairs the integrity of the cytoskeletal microtubule network and associated functions, and this leads to impaired uptake of nutrients and reduced parasite growth (Lacey, 1990). In the *E*. *multilocularis* genome, there are several beta-tubulin genes. The stem cells that develop in the germinal layer of *E*. *multilocularis* express mainly the *tub2* gene. The expressed Tub-2 protein does not efficiently bind to ABZ-sulfoxide, thus the stem cells are relatively resistant to the dosages of benzimidazoles used for treatment (Brehm and Koziol, 2014). There are additional factors, which could lead to the failure of benzimidazoles to act parasiticidally against *E*. *multilocularis*, such as the limited half-life of ABZ-sulfoxide in the host, and the restricted uptake of benzimidazoles by the parasite. In addition to beta-tubulin, enzymes of the energy metabolism were described as targets of benzimidazoles (Xiao et al., 1995), including fumarate reductase, an enzyme that is part of the malate dismutation pathway found in helminths like *Echinococcus* (Barrowman et al., 1984; Matsumoto et al., 2008). Unfortunately, these findings have not been further followed up. Studies based on electron microscopy showed that benzimidazole treatment of *E*. *multilocularis* metacestodes led to a rapid degeneration of the microtriches. This was shown for ABZ and its metabolites (Ingold et al., 1999), but also for other benzimidazoles such as fenbendazole and oxfendazole (fenbendazole-sulfoxide, see also Section 3.1). Interestingly, the microtriches cytoskeleton of *E*. *multilocularis* is composed of actin filaments and not microtubules (Küster et al., 2014b). Thus, benzimidazoles most likely affect other targets besides microtubules. A recent study in human patients showed that ABZ treatment increases the host immune response against the parasite (Ricken et al., 2017). To what extent this influences the efficacy of ABZ is unclear, and the crosstalk between chemotherapy and immunity should be further investigated. Possibly, benzimidazole treatment could slow down the general metabolism of the *E*. *multilocularis* metacestode, thus also reducing the release of potentially immunomodulatory components of the parasite. In this case, ABZ treatment would allow for partial restoration of the immune response against the parasite. However, even though there are studies published on the potential use of immunotherapy against AE (Boubaker et al., 2015; Wang et al., 2015, Wang et al., 2017, Wang et al., 2018a, Wang et al., 2018b), this basic question has not been addressed yet.

## 2 *In vitro* and *in vivo* models to study drug efficacy and drug targets in *E*. *multilocularis*

**Fig. 2:**
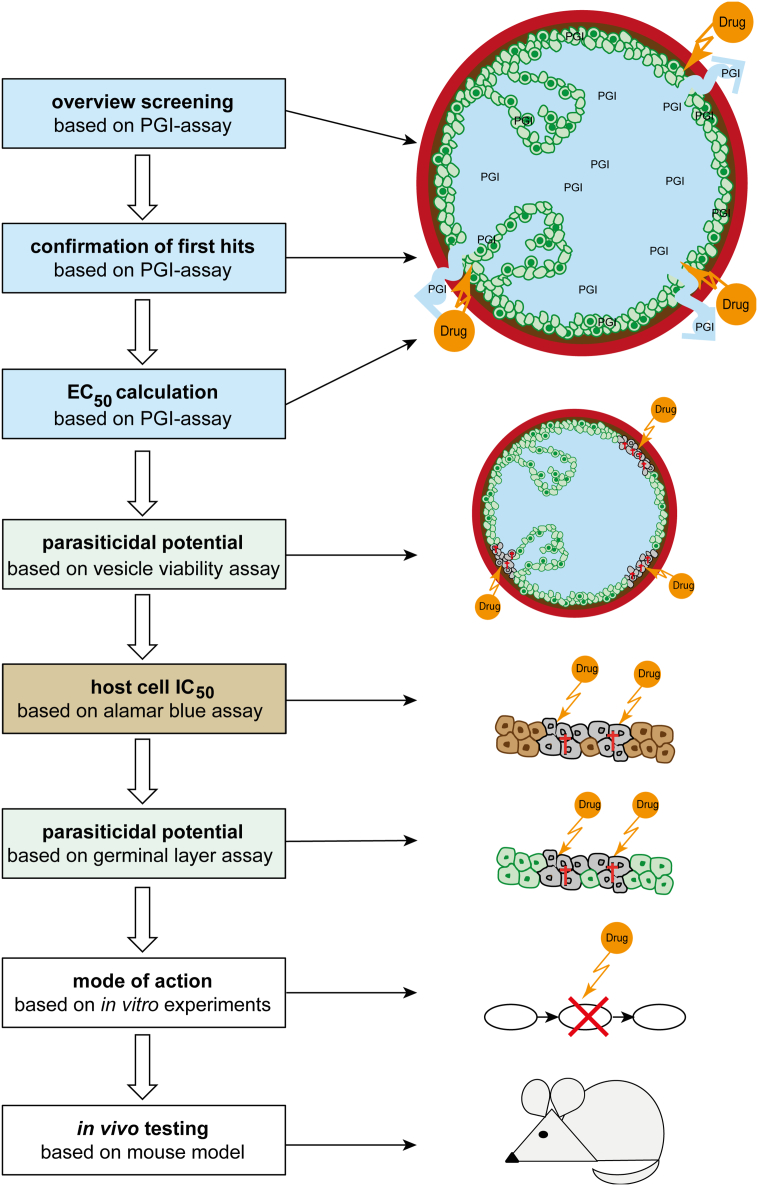
*In vitro* screening cascade of compounds against *E*. *multilocularis*. The three first steps of the screening are based on the PGI-assay that detects metacestode damage. Further, parasiticidal potential is assessed in the same model. If a potential therapeutic window can be identified by host cell toxicity assays, tests on isolated germinal layer cells are included to assess parasiticidal activity. The mode of action of a drug is further studied *in vitro* before studies in the mouse model are performed. Parasite cells are depicted in green, host cells in brown, dead cells in grey, drugs in orange. (For interpretation of the references to color in this figure legend, the reader is referred to the web version of this article.)

**Fig. 3:**
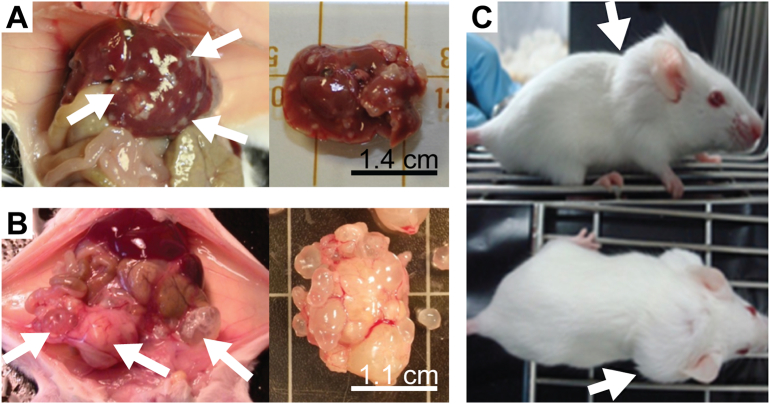
Different *in vivo* models for AE in mice. (A) Peroral infection with *E*. *multilocularis* eggs resulting in liver lesions. (B) Intraperitoneal infection with metacestode material resulting in peritoneal lesions. (C) Subcutaneous lesions visible from the outside (Küster et al., 2013a). Growing parasites are indicated by arrows.

*In vitro* culture of *E*. *multilocularis* metacestodes was reported as early as 1957 (Rausch and Jentoft, 1957). Other methods developed later (Hemphill and Gottstein, 1995; Jura et al., 1996) did not result in efficient production of metacestodes that would allow large-scale *in vitro* drug efficacy studies. In addition, these early drug studies relied solely on morphological observations rather than on objective assays for drug efficacy and parasite viability assessment (reviewed in Hemphill et al., 2010, Hemphill et al., 2014). Nowadays there are many tools to study *E*. *multilocularis*, reaching from a relatively easy to handle and standardized *in vitro* culture of metacestodes (Spiliotis et al., 2004), the publicly available genome and transcriptome (Tsai et al., 2013) and stem cell culture with methods for limited genetic manipulation (Mizukami et al., 2010; Spiliotis et al., 2008). This has rendered *E*. *multilocularis* the prime model for the study of diseases caused by the metacestode stage of cestodes in humans. Importantly, it has laid the basis for the development of objective methods for medium-throughput drug-testing *in vitro* (see Fig. 2, (Stadelmann et al., 2016)). A screening cascade to test up to several hundreds of compounds has been established as follows:

1. PGI-assay: physical impairment of *E*. *multilocularis* metacestodes is assessed in a first step by quantitatively measuring the vesicle fluid marker phosphoglucose isomerase (PGI) in the culture supernatant (Stadelmann et al., 2010). This quantitative assay allows determination of half-maximal effectivity (EC_50_) values and analyses of structure-activity relationships of tested compounds, such as recently shown for mefloquine and its derivatives by Rufener et al. (2018b). Similar approaches were also applied for related species like *T*. *solium* or *E*. *granulosus* (Cumino et al., 2012; Mahanty et al., 2011). However, the PGI-assay has also some drawbacks as it does not identify slow-acting drugs (such as benzimidazoles), and the published assay does not include any serum as a component of the culture medium to which drugs could bind (Beckmann et al., 2014).
2. Metacestode viability assay: to measure the viability of germinal layer cells within intact metacestodes, whole metacestodes treated with compounds of choice are assessed by alamar blue assay (Stadelmann et al., 2016). However, this test has a limited sensitivity, and does not allow for detection of single surviving stem cells, which could lead to parasite regrowth.
3. Cytotoxicity to mammalian cells: cytotoxicity to mammalian cells is measured for the compounds that were active in steps 1 and 2 to explore a potential therapeutic window. Commonly such tests are based on cell lines and conventional alamar blue assay. Ideally, several different mammalian or human cell lines should be used.
4. Stem cell viability: to identify if a compound is truly parasiticidal against *E*. *multilocularis*, the viability of isolated germinal layer cells (including stem cells) cultured *in vitro* can be assessed. As a readout of viability, ATP production is recorded by commercially available kits (Stadelmann et al., 2016). It should be noted that this test assesses the activity of drugs on isolated cells, which under natural conditions are embedded in the metacestode and protected by the laminated layer.
5. Mode of action: studies are performed on compounds with promising *in vitro* activities against *E*. *multilocularis* metacestodes and stem cells, but not against mammalian host cells. Assessments include electron microscopy to investigate structural alterations induced by those compounds (Rufener et al., 2018a). This method can indicate a potential mode of action or target organelle. Specific molecular targets can be identified by pull-down studies of immobilized drugs on a matrix, through which parasite extract is passed (Müller et al., 2008), or comparative monitoring of changes in the transcriptome, proteome, or metabolome of drug-treated *versus* non-treated parasites. Further validation of targets can include reverse genetic approaches based on RNAi, which is established in protoscoleces and stem cells of *E*. *multilocularis* (Mizukami et al., 2010; Spiliotis et al., 2010). Thus far, any other approaches for genetic manipulation of the parasite, such as by Crispr/Cas9 or lentiviral transfection systems, have not been successfully implemented.
6. *In vivo* activity against murine AE: compounds with promising *in vitro* efficacy and selective toxicity can be further evaluated using *in vivo* models. Voles are the natural intermediate hosts for *E*. *multilocularis*. Therefore, closely related laboratory mice and gerbils represent an ideal experimental model. However, *Mus musculus* is not a natural intermediate host for the parasite, and therefore future studies should also include the assessment of drug efficacy in natural hosts (*e*.*g*. dogs or captive monkeys). Mostly two experimental mouse infection models are applied: (i) the secondary infection model, in which mice are either intraperitoneally (Siles-Lucas and Hemphill, 2002) or subcutaneously (Küster et al., 2013a) infected with *E*. *multilocularis* metacestode suspension; (ii) the primary egg infection model (Stettler et al., 2004), in which mice are orally infected by gavage of *E*. *multilocularis* eggs, thus representing the natural route of infection (Fig. 3). Drug treatments are initiated 4–6 weeks post-infection, and the duration of treatment is variable, depending on the route of drug application, frequency of dosing, and the properties of the compounds used. Preferentially compounds are applied through the oral route, as this is the least invasive procedure and can be done on a daily basis if necessary. To facilitate this and to reduce the stress induced on the experimental animal, Küster et al. proposed to formulate drugs in honey to promote voluntarily ingestion of the compounds (Küster et al., 2012b), but this approach is not feasible for all compounds (own observations). The *in vivo* mouse model for drug testing was improved and standardized over the last years, by including ultrasound monitoring for non-invasive surveillance of treatment efficacy over time (Gorgas et al., 2017; Huang et al., 2018; Hübner et al., 2010; Küster et al., 2013a). At the end of treatment, the final parasite mass (for secondary infection), or lesion number and size (for primary infection) is assessed upon necropsy and compared to placebo-treated animals. For the secondary infection model, determination of the parasite weight was proven to be as accurate as non-invasive imaging approaches, and was shown to provide a clear readout of *in vivo* drug efficacy against AE (Gorgas et al., 2017). For the primary infection model, a PCR-based assessment for determining the presence or absence of *E*. *multilocularis* lesions in liver samples has recently been introduced (Rufener et al., 2018b). Future refinements should include also the quantitative assessment of such samples.

Pharmacokinetic analyses have been rarely applied in the murine AE model until to date. However, this is a critical step, as drugs that are active *in vitro* might not reach the parasite tissue *in vivo* at the needed concentrations. This might explain the discrepancy between promising *in vitro* and failing *in vivo* efficacy. Measurement and improvement of drug levels in treated animals is an important step to bring potential drugs a step closer to clinical application. A very recent study on mefloquine against AE has included such pharmacokinetic analyses (Rufener et al., 2018b)).

7. Interdisciplinary exchange: In order to ensure that potentially promising results on novel treatment options obtained in the laboratory will also reach those involved in the therapy of AE, and potentially also affected patients, respective findings should be directly communicated to clinicians and respective comments and feedback should be included in further studies. Thus, it is important to maintain corresponding contacts between clinicians and researchers and provide information in a timely fashion. Moreover, findings from trials in mice with AE should also be better communicated to veterinary clinicians, as also dogs and captive monkeys increasingly suffer from AE (Bacciarini et al., 2004; Brack et al., 1997; Brunet et al., 2015; Deplazes and Eckert, 2001; Frey et al., 2017; Rehmann et al., 2003). Naturally or experimentally infected dogs or naturally infected captive monkeys could represent another good model for the further study of compounds that were active in rodent models.

## 3 Drug repurposing – a viable strategy for the discovery of novel compounds to treat AE

The relatively low-level expected market return will render investments for the development of novel treatment options for AE on part of the pharmaceutical industry unlikely to happen. Moreover, diagnosis of AE is a difficult task, and treatment and follow-up monitoring of treatment success are time-consuming and complex undertakings requiring good medical facilities and financial resources that are normally not available in underdeveloped countries. As a consequence, AE is also not prioritized by foundations and private-public partnership organizations devoted to better management of neglected tropical diseases, since funding is restricted. Thus, the task of identifying novel compounds with activity against AE is left to the academic world, and the focus of this small community performing research on AE has been clearly on drug repurposing. For this, the plethora of drugs that are currently on the market, or that are being developed for other indications, are exploited for the purpose of identifying novel treatment options for AE. Repurposed drugs include mostly broad-spectrum anti-infective drugs (3.1, 3.2), and drugs that inhibit cellular proliferation such as anti-cancer compounds (Section 3.3). In addition, natural products (Section 3.4) have been increasingly investigated. In most studies, only relatively small numbers of compounds were evaluated, even though for *E*. *multilocularis* small drug libraries could be screened thanks to the establishment of an optimized *in vitro* culture and a standardized whole-organism-based screening cascade (see Section 2).

### 3.1 Repurposing of anti-infective agents for the treatment of AE

The reason for repurposing anti-infective compounds against AE results from the fact that *E*. *multilocularis* metacestodes are highly adapted to a parasitic lifestyle, must scavenge nutrients from their host, and exert considerable immunomodulation. These are features they share with many other infectious pathogens. Therefore, drugs that affect other pathogens could also be efficacious against *E*. *multilocularis*.

Early animal experimentation studies in rodents (see Supplementary Table 1) demonstrated some activity of the anti-parasitic lucanthone (Lubinsky, 1969a, Lubinsky, 1969b, Lubinsky, 1969c), the anti-viral isoprinosine (Sarciron et al., 1995, Sarciron et al., 1992, Sarciron et al., 1991) and derivatives of the anthelmintic piperazine (Mikhaĭlitsyn et al., 1994, Mikhaĭlitsyn et al., 1991). Also tested, but not effective, were the anti-parasitics tiguvon, neguvon and dimercaptosuccinate (Lubinsky, 1969a, Lubinsky, 1969b, Lubinsky, 1969c), trimethoprim, pyrimethamine, metronidazole, diethylcarbamizine, hycanthone, Hoechst S-201 and Hoechst S-616 (Lubinsky et al., 1971), and ivermectin (Inaoka et al., 1987). *In vivo* treatments with alpha-difluoromethylornithine against secondary AE were also ineffective (Miyaji et al., 1993).

The impact of further anti-infective compounds was more thoroughly studied *in vitro* before respective studies in mice were performed (see Supplementary Table 2). These include the current drug in use against AE, ABZ and its metabolites ABZ-sulfoxide and ABZ-sulfone (Ingold et al., 1999), but also other benzimidazoles such as fenbendazole and methiazole (Küster et al., 2014b; Reuter et al., 2006). A more detailed review on the different benzimidazoles tested against echinococcosis is given by Siles-Lucas et al. (2018). Further tested anti-infective agents are nitazoxanide and derivatives (Stadelmann et al., 2010; Stettler et al., 2003), the antibiotic clarithromycin (Mathis et al., 2005), the anti-fungals amphotericin B (Reuter et al., 2003b), itraconazole and caspofungin (Reuter et al., 2006), the anti-parasitic miltefosine (Reuter et al., 2006), the antibiotics rifampicin and trimethoprim/sulfamethoxazole (Reuter et al., 2006), thioureides (Müller et al., 2009), antiprotozoal pentamidines such as the thiopene-di-guanidino compound DB1127 (Küster et al., 2013b; Stadelmann et al., 2011), and a variety of anti-malarials (see Section 3.2). In addition, a library of 400 anti-infective compounds from the Medicines for Malaria Venture (MMV) Pathogen box has recently been screened (Rufener et al., 2018a). In the abovementioned studies, (a) the benzimidazoles ABZ, its two metabolites, as well as fenbendazole, and methiazole, (b) nitazoxanide, (c) amphotericin B and itraconazole, (d) clarithromycin, (e) DB1127, and (f) buparvaquone were among the most effective compounds against *E*. *multilocularis* metacestode vesicles *in vitro*. On each of these active compounds, more information is given below:

(a) Benzimidazoles: further investigations showed that fenbendazole exhibits similar activities as ABZ when administered to experimentally infected mice (Küster et al., 2014b). The benzimidazoles ABZ and fenbendazole are supposed to act with an identical mode of action on microtubules, and thus inhibit a variety of cellular functions related to the integrity of the cytoskeleton, like the microtubule-dependent uptake of glucose (Lacey, 1990). Improved *in vivo* efficacy of fenbendazole against AE could possibly be achieved by employing the pro-drug febantel, which is better absorbed, and this would result in a prolonged half-life of the active fenbendazole-sulfoxide (also known as oxfendazole). Methiazole is a newer benzimidazole, which is structurally related to ABZ. Methiazole was able to destroy *E*. *multilocularis* metacestodes *in vitro* at similar concentrations as ABZ (Reuter et al., 2006). However, also this drug acted only parasitostatic, since upon removal of methiazole, the parasite resumed growth.
(b) Nitazoxanide: the broad-spectrum anti-parasitic thiazolide nitazoxanide has reported anti-parasitic, anti-bacterial, and anti-viral activities (Hemphill et al., 2006). Nitazoxanide was active against metacestodes of *E*. *multilocularis in vitro* and applied orally by gavage also in experimentally infected mice against AE (Stettler et al., 2004, Stettler et al., 2003). However, ABZ-nitazoxanide combination treatment was more effective than ABZ alone, which is not being caused by synergistic modes of action, but due to competing metabolization through cytochrome P450 enzymes. This leads to a prolonged presence of ABZ-sulfoxide in the serum (Stettler et al., 2004). To investigate the structure-activity relationship and possibly improve nitazoxanide for application against AE, 29 nitazoxanide derivatives were tested against *E*. *multilocularis* metacestodes *in vitro* using the PGI-assay (Stadelmann et al., 2010). Enhanced anti-parasitic activity was noted for some nitro compounds similar to nitazoxanide, but also for compounds with halogenated thiazole and salicyl moieties, and extensive morphological damage was noted already after 5 days of treatment (Stadelmann et al., 2010). However, these promising results were not followed-up, since the company marketing nitazoxanide refused to further collaborate. In human AE patients, nitazoxanide therapy was ineffective (Kern et al., 2008; Tappe et al., 2009).
(c) Amphotericin B and itraconazole: the anti-fungal drug amphotericin B inhibited the growth of *E*. *multilocularis* metacestodes *in vitro*, and even in human patients *in vivo* (Reuter et al., 2003b, Reuter et al., 2003a). However, amphotericin B acts only parasitostatic, it needs to be applied intra-venously, and the drug is nephrotoxic, which makes it unsuitable for prolonged use. Thus, amphotericin B was only used for salvage treatment. Nevertheless, prolonged application of amphotericin B may be feasible in selected cases (Reuter et al., 2003a). Itraconazole has a similar mode of action as amphotericin B, as it inhibits sterol biosynthesis. In contrast to amphotericin B, it is orally applied, there is extensive clinical information, including long-term use, and there is no nephrotoxicity expected. However, *in vitro* tests showed that its action is delayed in comparison to amphotericin B and also itraconazole is not parasiticidal against *E*. *multilocularis* metacestodes (Reuter et al., 2006).
(d) Clarithromycin: the macrolide antibiotic clarithromycin was identified by a target-based *in silico* approach (Mathis et al., 2005). Clarithromycin inhibits protein synthesis in bacteria by binding to a specific site of the large subunit rRNA (Rodriguez-Fonseca et al., 1995). In contrast to bacteria, cytoplasmic and mitochondrial rRNAs of higher eukaryotes carry a guanine at position 2058, and this confers resistance of eukaryotic cells to macrolide antibiotics. The mitochondrial rRNA of *E*. *multilocularis*, similar to bacteria, carries an adenine at sequence position 2058, which predicts susceptibility (Sander et al., 1997), while in the nucleus-encoded rRNA, this position is filled by a guanine, like in higher eukaryotes. *In vitro* exposure of *E*. *multilocularis* metacestodes with clarithromycin resulted in severely impaired growth and altered morphology in the germinal layer. However, no *in vivo* studies on clarithromycin have been reported so far.
(e) DB1127: a screening of a small panel of di-N-aryl-diguanidino compounds against *E*. *multilocularis* metacestodes *in vitro* revealed that only those molecules with a thiophene core group were active against metacestodes, while furans were not (Küster et al., 2013b; Stadelmann et al., 2011). The most active compound in these studies was DB1127. DB1127 was effective against AE in mice when administered intraperitoneally but not when applied orally (Küster et al., 2013b). Therefore, thiophene-diguanidino derivatives with improved oral bioavailability should be further developed and investigated.
(f) Buparvaquone: Rufener et al. (2018a) screened the MMV pathogen box, which contains compounds against various infectious diseases, such as tuberculosis, malaria, dengue, and toxoplasmosis, against *E*. *multilocularis* metacestodes *in vitro*. One of the compounds with strong activity was buparvaquone (Rufener et al., 2018a). Buparvaquone is already marketed for treatment of theileriosis in cattle where it is administered as an intramuscular injection. It was also shown to be active against a range of other parasites including *Toxoplasma*, *Neospora*, and *Leishmania*, and was thus further investigated. In protozoan parasites, buparvaquone is known to act as a cytochrome *bc*1 inhibitor, thus it interferes in oxidative phosphorylation in the mitochondrion. Rufener et al. (2018a) showed that this also occurs in isolated germinal layer cells of *E*. *multilocularis*, and in metacestodes the drug has a strong impact on the ultrastructure of the mitochondria. However, oral application of buparvaquone in a secondary mouse infection model for AE did not result in diminished parasite mass. Most likely the bioavailability of the drug needs to be improved, and formulations that allow a more targeted delivery to the parasite need to be developed. In this context it is interesting to note that helminths, including cestodes, harbor an additional energy generating pathway (the malate dismutation pathway). This pathway is also active under microaerobic and anaerobic conditions, and it is thus not affected by buparvaquone. Clearly, the energy metabolism pathways of these parasites represent interesting drug targets that should be further studied.

### 3.2 Repurposing of anti-malarials for the treatment of *E*. *multilocularis*

A range of anti-malarial compounds has been reported to exhibit considerable activity against helminths (Panic et al., 2014). *In vitro* studies were carried out with the following compounds (see Supplementary Table 2):

(a) Artemisinins: *in vitro* treatment with the artemisinin-derivatives dihydroartemisinin and artesunate exhibited promising results (Spicher et al., 2008b). However, respective therapies in mice experimentally infected with *E*. *multilocularis* during 6 weeks did not affect parasite growth, also not when combined with ABZ.
(b) Ozonids: to identify further artemisinin derivatives with possibly improved activity against *E*. *multilocularis*, a series of artemisinin-peroxides (ozonids) were comparatively assessed *in vitro* (Küster et al., 2014a). The three ozonids OZ401, OZ455, and OZ491 containing an amino-propylether substructure were the most active, with IC_50_ values ranging from 11 to 14 μM and no cytotoxicity against various mammalian cell lines at these concentrations. Ozonids were not further pursued, since the concentrations required for anti-echinococcal activity cannot be achieved in animals or humans.
(c) Mefloquine: the most intensely studied anti-malarial drug against helminths, including *E*. *multilocularis*, is mefloquine. Mefloquine was active against mice infected with young or adult stages of *Schistosoma mansoni* or *S*. *japonicum* (Keiser et al., 2009a; Manneck et al., 2010), against *Opisthorchis viverrini in vitro* and in infected hamsters (Keiser et al., 2009b) as well as against larval and adult stages of *Brugia patei* and *B*. *malayi in vitro* (Walter et al., 1987). *In vitro* treatment of *E*. *multilocularis* metacestodes resulted in detachment of large parts of the germinal layer from the inner surface of the laminated layer within a few hours and concentration-dependent PGI release (Küster et al., 2011). Intraperitoneal application of mefloquine in secondarily-infected mice (25 mg/kg, twice a week) was equally effective as orally applied ABZ (200 mg/kg/day) (Küster et al., 2011). Oral application of mefloquine, however, was only active when a higher dose (100 mg/kg, twice per week) was applied (Küster et al., 2015). Recently, egg-infected mice were shown to benefit from oral mefloquine treatment (100 mg/kg, twice per week) as well, with significantly fewer parasite lesion numbers found in the liver than with ABZ- or mock-treatment (Rufener et al., 2018b). Further, this study also investigated the plasma levels in mice, and found that these were at a steady state level of C_min_ = 1.15 mg/L and C_max_ = 2.63 mg/L. This is in a range which could be achieved by a malaria-prophylactic dose of mefloquine in humans. Thus, this already licenced drug could possibly be active in salvage treatment against human AE. The major drawback of mefloquine is its described neuropsychiatric side-effects. To possibly identify derivatives with lower toxicity, or higher activity than mefloquine, several mefloquine-derivatives were comparatively assessed *in vitro*, but none of them showed an improved profile when compared to mefloquine (Rufener et al., 2018b).
(d) MMV665807: Other anti-malarial drugs with *in vitro* efficacy against *E*. *multilocularis* metacestodes were identified by repurposing of the open-source malaria box from MMV (Stadelmann et al., 2016). This library contains 400 commercially available chemicals that show *in vitro* activity against *Plasmodium falciparum*. Screening against *E*. *multilocularis* metacestodes revealed that the compound MMV665807 exhibited selective activity against *E*. *multilocularis* metacestodes and germinal layer cells. MMV665807 is a salicylanilide-derivative, similar to the already commercially available niclosamide and nitazoxanide. Niclosamide is active against adult stage cestodes (Tanowitz et al., 1993) and also efficacious against various cancer cells *in vivo* and *in vitro* (Liu et al., 2016). Unfortunately, when assessed in experimentally infected mice, neither oral nor intraperitoneal application of MMV665807, resulted in any reduction of metacestode burden (Stadelmann et al., 2016). However, different formulations of MMV665807 are currently prepared, which are designed to achieve increased plasma levels, and they will be assessed *in vitro* and *in vivo*.

### 3.3 Repurposing of anti-cancer drugs for the treatment of AE

An alternative approach for drug repurposing against AE is to study the effects of anti-cancer (or anti-proliferative) drugs. *E*. *multilocularis* metacestodes and malignant tumours have several features in common: they have an unlimited proliferative capacity and have the potential to form metastases, they modulate the immune response, they secrete proteolytic enzymes to reach their target sites or organs, they express similar cell cycle regulators such as 14-3-3 protein, and they induce angiogenesis. As for anti-infective drugs, also early studies on anti-proliferative drugs (see Supplementary Table 1) were exclusively performed in animal models, namely intraperitoneally infected rodents. These included cyclophosphamide, hydroxyurea, vinblastine, vincristine, demecolcine and dactinomycine (Lubinsky, 1969b; Lubinsky et al., 1971), mitomycin C (Novak, 1990), and doxorubicin (Liance et al., 1993). While mitomycin C, cyclophosphamide and doxorubicin appeared to exhibit good activities (see below), results were less promising for demecolcine, and dactinomycine. Doxorubicin, a DNA-interacting drug widely applied in the treatment of cancers, was active in mice only when bound to polyisohexylcyanoacrylate nanoparticles. In contrast, free doxorubicin or unbound nanoparticles had no anti-parasitic effects (Huang et al., 2018; Liance et al., 1993). Unfortunately, doxorubicin is also known to induce massive adverse side effects, and therefore this treatment approach was not further pursued. Cyclophosphamide had a high efficiency in the AE mouse model with only a single application of the drug (Lubinsky, 1969c). However, no further studies reported on this compound.

Later studies on anti-cancer drugs against *E*. *multilocularis* were not exclusively based on the mouse model, but also more detailed *in vitro* analyses against metacestodes were performed (Supplementary Table 2). These studies reported on (a) isoflavonoids and genistein (Naguleswaran et al., 2006), (b) 2-methoxyestradiol (Spicher et al., 2008a), (c) methotrexate, navelbine and vincristine (Huang et al., 2018; Hübner et al., 2010), (d) kinase inhibitors (Gelmedin et al., 2010, 2008; Hemer and Brehm, 2012; Schubert et al., 2014), (f) metallo-organic ruthenium complexes (Küster et al., 2012a), (g) the proteasome inhibitor bortezomib (Stadelmann et al., 2014), and (h) taxanes (Huang et al., 2018).

(a) Isoflavonoids: isoflavonoids are anti-infective molecules that are synthesized by plants. Genistein, a major component of soy, is active against breast, prostate, skin, and colon cancer cell lines, and it stimulates the synthesis of TGF-β, which itself inhibits cancer cell proliferation (Messina, 1999). Genistein acts on several signalling pathways, including kinases (tyrosine kinase, MAP kinase, ribosomal S6 kinase) and can bind to the estrogen receptor-b (Pike et al., 1999). *In vitro* assessment of genistein and non estrogen-binding derivatives against *E*. *multilocularis* metacestodes revealed that they were all active (Naguleswaran et al., 2006). Possibly, these compounds could interfere in *E*. *multilocularis* signalling, but the molecular mechanisms have not been elucidated. In contrast, the estrogen receptor-alpha antagonist tamoxifen, which is active against primary breast cancer, was moderately active against *E*. *multilocularis in vitro* (Stadelmann et al., 2014). The anti-echinococcal efficacy of isoflavonoids or tamoxifen has not been assessed *in vivo* to date.
(b) 2-Methoxyestradiol: this is an endogenous metabolite of estrogen. 2-methoxyestradiol (2-ME) induces severe and dose-dependent damage to *E*. *multilocularis* metacestodes *in vitro*. In the murine model of AE, however, 2-ME was not effective (Spicher et al., 2008a).
(c) Methotrexate, navelbine, vincristine: the cytostatic agents methotrexate, navelbine, and vincristine were used for *in vitro* treatments of *E*. *multilocularis* and subsequently injected into gerbils. While navelbine and vincristine had a slight negative impact on parasite development, methotrexate led to a massive increase of parasite growth (Hübner et al., 2010).
(d) Kinase inhibitors: signal transduction, cell growth and differentiation are largely dependent on the activity of a multitude of protein kinases, especially serine/threonine and tyrosine kinases. Such kinases are also crucial regulators of tumor cell growth. Among the most promising drug targets identified in the *E*. *multilocularis* genome (Tsai et al., 2013), protein kinases take the most prominent role. These kinases fulfill crucial functions in signal transduction, growth regulation, differentiation, and host-parasite communication, allowing the parasite to react to changes at the host-parasite interface. The signalling receptors expressed by *E*. *multilocularis* metacestodes include nuclear hormone receptor, TGF-receptor, insulin receptor, epidermal growth factor receptor and fetal growth factor receptor, and these receptors can be activated by either parasite-ligands or the corresponding host-derived homologues (Brehm, 2010b; Brehm and Koziol, 2017; Spiliotis et al., 2008). ML3403 and SB202190 are inhibitors of p38 mitogen-activated protein kinases (MAPK) developed for cancer-treatment (Bellei et al., 2012), and they were shown to similarly also act on *E*. *multilocularis in vitro* (Gelmedin et al., 2008). Other kinase inhibitors that were assessed against *E*. *multilocularis* vesicles *in vitro* are the Raf-inhibitor sorafenib and the MEK1/2 inhibitor PD184352, which inhibited vesicle growth, but failed to exert parasticidal activity (Gelmedin et al., 2010). The ABL-like kinase inhibitor imatinib, one of the first FDA approved kinase inhibitors for anti-cancer treatment, exhibited dose-dependent efficacy against *E*. *multilocularis* metacestodes, protoscoleces and stem cell cultures *in vitro* (Hemer and Brehm, 2012). In the secondary AE mouse model, however, the related and also licenced ABL-like kinase inhibitor nilotinib failed to be active (Joekel et al., 2018). Another kinase inhibitor thoroughly investigated *in vitro* against *E*. *multilocularis* metacestodes is the polo-like kinase inhibitor BI2536. BI2536 blocked *E*. *multilocularis* vesicle formation from germinal cell cultures (Schubert et al., 2014). In addition, BI2536 eliminated the stem cell population from mature metacestode vesicles *in vitro*, resulting in parasite tissue that was no longer capable of proliferation. However, this inhibitor was not further investigated in experimentally infected mice. A serine/threonine kinase inhibitor, which was assessed *in vitro* and *in vivo*, is everolimus. However, the drug failed to lead to a reduction in parasite mass in infected mice when compared to mock-treated animals (Joekel et al., 2018). Thus, a series of kinase inhibitors that are candidate drugs (or are in use) for cancer treatment exhibit profound inhibitory properties on *E*. *multilocularis in vitro*, but positive findings in the mouse model are lacking to date.
(e) Metallo-organic ruthenium complexes: these are a rather novel class of anti-cancer compounds that were also shown to exhibit interesting anti-microbial properties, including activities against bacteria, trypsanosomatids, and apicomplexan parasites (Basto et al., 2017; Corrêa et al., 2016; Macedo et al., 2016; Southam et al., 2017). Various η6-areneruthenium(II) phosphite complexes were tested *in vitro* for their activity against *E*. *multilocularis* metacestodes and some of them were highly active (Küster et al., 2012a). They all yielded also high cytotoxicity against rat hepatoma cells, but little for other non-cancer cells. This indicates a potential for ruthenium compounds against AE, but corresponding *in vivo* studies are lacking.
(f) Bortezomib: the first screening of a commercially available drug library against *E*. *multilocularis* metacestodes was based on the 426 compounds included in a FDA-approved drug library (Stadelmann et al., 2014). This library was comprised of drugs against various diseases, including infectious diseases and cancer. Upon screening of this library and further *in vitro* studies, the proteasome inhibitor bortezomib was identified as the most interesting compound. Its EC_50_ against metacestodes was 0.6 μM, and it led to an accumulation of ubiquinated proteins and unequivocally parasite death. Zymography assays applying *E*. *multilocularis* extracts demonstrated bortezomib-mediated inhibition of the proteasome subunit beta 5 of *E*. *multilocularis*. Treatment of secondarily infected mice with bortezomib led to slightly reduced parasite weight, but this was not statistically significant, and it induced adverse effects such as diarrhea and neurological symptoms (Stadelmann et al., 2014). Nevertheless, this study identified the proteasome as a drug target in *E*. *multilocularis* metacestodes, which could be inhibited in the future by employing other proteasome inhibitors.
(g) Taxanes: in a recent study by Huang et al. (2018), *E*. *multilocularis* metacestodes were treated *in vitro* by various cytostatic agents. Upon treatment with docetaxel, and to a lesser extent also paclitaxel, further *in vitro* metacestode vesicle formation was inhibited. *In vitro* treated metacestodes were re-injected into gerbils and followed up by magnetic resonance imaging and positron emission tomography with the 2-deoxy-2-18F-fluoro-d-glucose tracer. Hereby, no more parasite growth was observed within 3 months after treatment with docetaxel, paclitaxel, or navelbine (Huang et al., 2018). After 5 months, there was limited regrowth in the docetaxel-treated group only at the lowest tested concentration, but paclitaxel and navelbine failed to prevent metacestode regrowth. The taxanes paclitaxel and docetaxel are FDA-approved prostate cancer drugs. They both inhibit microtubule disassembly, and therefore block cells in the G2/M phase of the cell cycle, which leads to apoptosis. Whether the same mechanism of action applies for *E*. *multilocularis*, needs to be further investigated. Certainly, taxanes should receive further attention as future drugs against AE.

### 3.4 Repurposing of natural products for the treatment of AE

A continuously growing list of natural products and plant extracts has been, and still is, tested for a potential application in the treatment of *E*. *granulosus* infection (Siles-Lucas et al., 2018). In contrast, the number of extracts of natural products assessed for activity against *E*. *multilocularis* is still sparse. In combination with ABZ, thymol, a monoterpene which is a major component of essential oils of several plant species including oregano and thyme, was reported to have considerable activity against *E*. *multilocularis* protoscoleces and metacestodes (Albani Clara and Elissondo María, 2014). In secondarily infected mice, combined ABZ/thymol treatment for as few as 20 days resulted in a significantly reduced parasite weight compared to ABZ or thymol treatments alone (Albani et al., 2015). However, we have not been able to confirm these *in vitro* efficacy results in our own laboratory employing the *E*. *multilocularis* protoscolex movement assay (Ritler et al., 2017) or PGI-assay (Stadelmann et al., 2010) (unpublished data). Another study reported on the positive effects of osthole (a substance found in *Cnidium monnieri*) in *E*. *multilocularis* infected mice (Yuan et al., 2016).

## 4 Where to go from here

Several strategies should be followed to reach the goal of improving treatment efficacy in AE. Even though benzimidazoles have drastically improved the life-expectancy of echinococcosis patients, there are still several important drawbacks. More recent studies suggest to improve the absorption and oral bioavailability of these drugs by developing new formulations such as benzimidazole salt formulations (Cirilli et al., 2017), nanocrystals (Pensel et al., 2018), liposome formulations (Li et al., 2016; Lv et al., 2013) or chitosan microspheres (Abulaihaiti et al., 2015). Going down this path would shorten the duration of therapy and thus avoid adverse side effects.

However, as shown here, considerable efforts have been undertaken to discover alternatives to benzimidazoles for the treatment of AE. An increasing number of studies have been performed *in vitro*, thanks to the development of suitable culture techniques for *E*. *multilocularis* metacestodes and germinal layer cells. Most notably, few compounds with promising *in vitro* characteristics have actually been reported to be assessed *in vivo*, while most others have not been followed up. Reasons for this could lie in (i) lack of project financing, (ii) lack of specificity and toxicity of the compound, or (iii) lack of interest in publishing negative findings, which is, unfortunately, a commonly observed fact. In any case, following studies in small laboratory models, most substances were not further pursued, even though promising results were produced. This bottleneck can be explained by financial constraints, which have hindered further studies in larger animals and/or humans.

Nevertheless, two drugs (amphotericin B and nitazoxanide) have been applied in human AE patients, thanks to the efforts of academic institutions. However, while amphotericin B was useful only as a salvage treatment, nitazoxanide was shown to be ineffective.

Probably the most promising compound for further application in AE patients is the anti-malarial mefloquine. When applied orally at a dosage of 100 mg/kg (Küster et al., 2015), the drug halted parasite growth, although it did not prove to be acting in a parasiticidal manner. Serum levels in mice at this effective dosage corresponded roughly to the levels that are achieved during a prophylactic regimen in humans (Rufener et al., 2018b). However, up to date there is a justified reluctance to the long-term application of mefloquine in AE patients, due to potential neurological side-effects (Tickell-Painter et al., 2017).

Based on their strong activity against *E*. *multilocularis* metacestodes *in vitro*, parasiticidal potential *in vitro*, marketing status and the potential of improved formulations to reach activity in the AE mouse models, the following compounds were identified as promising and are further investigated: MMV665807, buparvaquone, and taxanes. Nevertheless, novel treatment options that act *in vivo* parasiticidally are still lacking. Thus, further efforts should focus on the screening of additional drug libraries and/or generating derivatives of these active compounds with improved bioavailability and pharmacokinetic properties. In addition, biochemical and molecular studies are needed to identify relevant drug targets, and to understand the mechanisms of action that are relevant for exerting parasiticidal activity (Hemphill et al., 2014). Combining drugs with different mechanisms of action could produce synergistic effects and improve treatment efficacy.

An additional promising strategy could be to further investigate, and exploit, the metabolic requirements of *E*. *multilocularis* metacestodes. Cestodes are highly adapted to a parasitic life style, and they lack essential genes and pathways for the synthesis of pyrimidines, purines, amino acids, and other metabolites. In addition, genes for fatty acid and cholesterol *de novo* synthesis are largely missing (Tsai et al., 2013). Thus, in order to fulfill their metabolic needs, metacestodes are forced to scavenge these metabolites from their host, and transcription of genes coding for respective enzymes involved in uptake and transport was shown to be upregulated in the metacestode stage. These auxotrophies could be exploited for the development of novel therapeutic options. In addition, the malate dismutation pathway, present in helminths but not in mammals, provides the unique opportunity to target these parasites. In this pathway, the anaerobic NADH-fumarate reductase system is a predominant component in the energy metabolism of *E*. *multilocularis*, and the development of compounds that specifically inhibit this system, possibly synergistically with other known drugs that act on oxidative phosphorylation, could result in efficacious strategies for the treatment of AE (Matsumoto et al., 2008).

However, while much should and could be done, finances for building up a research program on novel drugs for echinococcosis are difficult to acquire, and funding on this topic is not being regarded as a priority, neither by private nor public authorities. Hopefully, this will change in the near future.

## Appendix A Supplementary data

Table S1 List of compounds tested against *E*. *multilocularis* metacestodes *in vivo*. Treatment regimens, outcome of the studies and references are specified. Compounds are given in alphabetical order. p.o. = *per os*, i.p. = intraperitoneal, s.c. = subcutaneous, i.v. = intravenous, n/a = not applicable. Table S2 List of compounds tested against *E*. *multilocularis* metacestodes *in vitro*. Outcome of the studies and references are given. Note that the upper part of the table shows libraries, which were screened for activity against *E*. *multilocularis*. Below, compounds are given in alphabetical order. Image 1
